# Successful implementation of an enhanced recovery after surgery programme for elective colorectal surgery: a process evaluation of champions’ experiences

**DOI:** 10.1186/s13012-015-0289-y

**Published:** 2015-07-17

**Authors:** Lesley Gotlib Conn, Marg McKenzie, Emily A. Pearsall, Robin S. McLeod

**Affiliations:** Evaluative Clinical Sciences, Trauma, Emergency and Critical Care Research Program, Sunnybrook Research Institute, 2075 Bayview Ave., Room K3W-28, Toronto, ON M4N 3M5 Canada; Department of Surgery, Mount Sinai Hospital Joseph and Wolf Lebovic Health Complex, 600 University Avenue, Toronto, ON M5G 1X5 Canada; Department of Surgery, and Institute of Health Policy, Management and Evaluation, University of Toronto, Toronto, ON M5T 1P5 Canada

**Keywords:** Enhanced recovery after surgery, Implementation, Process evaluation, Qualitative research, Normalization process theory

## Abstract

**Background:**

Enhanced recovery after surgery (ERAS) is a multimodal evidence-based approach to patient care that has become the standard in elective colorectal surgery. Implemented globally, ERAS programmes represent a considerable change in practice for many surgical care providers. Our current understanding of specific implementation and sustainability challenges is limited. In January 2013, we began a 2-year ERAS implementation for elective colorectal surgery in 15 academic hospitals in Ontario. The purpose of this study was to understand the process enablers and barriers that influenced the success of ERAS implementation in these centres with a view towards supporting sustainable change.

**Methods:**

A qualitative process evaluation was conducted from June to September 2014. Semi-structured interviews with implementation champions were completed, and an iterative inductive thematic analysis was conducted. Following a data-driven analysis, the Normalization Process Theory (NPT) was used as an analytic framework to understand the impact of various implementation processes. The NPT constructs were used as sensitizing concepts, reviewed against existing data categories for alignment and fit.

**Results:**

Fifty-eight participants were included: 15 surgeons, 14 anaesthesiologists, 15 nurses, and 14 project coordinators. A number of process-related implementation enablers were identified: champions’ belief in the value of the programme, the fit and cohesion of champions and their teams locally and provincially, a bottom-up approach to stakeholder engagement targeting organizational relationship-building, receptivity and support of division leaders, and the normalization of ERAS as everyday practice. Technical enablers identified included effective integration with existing clinical systems and using audit and feedback to report to hospital stakeholders. There was an overall optimism that ERAS implementation would be sustained, accompanied by concern about long-term organizational support.

**Conclusions:**

Successful ERAS implementation is achieved by a complex series of cognitive and social processes which previously have not been well described. Using the Normalization Process Theory as a framework, this analysis demonstrates the importance of champion coherence, external and internal relationship building, and the strategic management of a project’s organization-level visibility as important to ERAS uptake and sustainability.

## Introduction

Enhanced recovery after surgery (ERAS) is a multimodal evidence-based approach to patient care that has become the standard in elective colorectal surgery [1, 2]. ERAS is an interprofessional, goal-directed programme that begins for patients in the preoperative period and extends through hospital discharge (Table 1). ERAS programmes have been developed globally [3–5] with the aim of decreasing perioperative stress, improving pain management and gut dysfunction, and minimizing postoperative complications which will then lead to hastened patient recovery and reduced time in hospital [6–9]. Since the early 1990s, ERAS programmes have been shown to significantly improve the quality of patient care in colorectal surgery leading to reductions in hospital length of stay and patient morbidity [10–12], as well as benefiting resource utilization [11]. Improved outcomes with ERAS programmes are not limited to colorectal surgery but are similarly found in orthopaedics and other surgeries [13–15].

**Table 1:** Summary of ERAS guideline recommendations

Preoperative	Preoperative counselling
	Reduced fasting duration
	Carbohydrate drinks
	No mechanical bowel preparation
Intraoperative	NSAIDS (non-steroidal anti-inflammatory drugs)
	± TEA (thoracic epidural analgesia)
	No abdominal drains
	No nasogastric tubes
	Multimodal pain management
	Thromboprophylaxis
	Surgical site infection (SSI) prophylaxis
	Goal-directed fluid management
	Normothermia
	TEA or intravenous (IV) Lidocaine
Postoperative	Fluid restriction
	Early removal of urinary catheters
	Gum chewing
	Early ambulation
	Early feeding
	Multimodal pain management

ERAS programmes represent a considerable change in practice for many surgical care providers. For example, where preoperative fasting was previously the norm, ERAS guidelines recommend that patients be allowed clear liquids up to 2–3 h prior to surgery. Early enteral feeding and early mobilization are noteworthy changes to postoperative care introducing solid food and walking on the morning following surgery. Among anaesthesiologists, ERAS recommendations for multimodal pain management and intraoperative fluid management are new and in some cases contentious. In addition to these specific practice changes, the delivery of ERAS is anchored in an interprofessional approach whereby the entire bundle of interventions is most optimally executed in coordination by the surgeons, nurses, anaesthesiologists, physiotherapists, dietitians, and non-clinical personnel whom surgical patients encounter in hospital.

Given the complexity involved in implementing a relatively high number of interventions simultaneously among many providers and across hospital services, a number of implementation barriers have been identified. While reports of protocol compliance are encouraging [16, 17], there is a general consensus that ERAS uptake has been relatively slow and inconsistent despite the strength of supporting evidence [17, 18]. Implementation challenges have been attributed to a variety of contextual factors such as perceived lack of resources, resistance to change among providers, and poor buy-in, all which impede uptake [19–21]. A number of studies have found protocol compliance in the postoperative period particularly challenging and have suggested structural reorganization and continuing staff education as solutions [17, 22–24]. ERAS sustainability is described more recently in the literature though our current understanding of the specific challenges is limited [25–27]. Quantitative measures have provided insight into the presence or absence of ERAS interventions over the long term; however, we currently know very little about the qualitative adaptive aspects [28] of the implementation process that might lead to sustainable ERAS practice.

## Background

In January 2013, we began a 2-year ERAS implementation for elective colorectal surgery in Ontario. The project was funded through a peer-reviewed competition held by the Council of Academic Hospitals of Ontario (CAHO), a non-profit association that is governed by a consortium of hospital executives to support strategic initiatives within academic hospitals in the province. In 15 academic hospitals, we implemented ERAS using a multifaceted approach involving both technical and adaptive features targeting clinical and socio-cultural outcomes [28]. The strategy included: identification and support of local champions in surgery, nursing, and anaesthesia; development of a community of practice [29]; audit and feedback on clinical performance [30]; development of pre-printed orders, staff reminders, and patient education materials; facilitation of communication, networking, and sharing best practices among disciplines and centres; and, support from hospital administration. It also included hiring a site coordinator for each participating hospital whose role was primarily clinical data collection. The full details of our knowledge translation and implementation strategy have been previously published [9].

On all accounts, the ERAS implementation project was successful with 2475 patients enrolled across 15 centres. Overall, compliance with guideline recommendations increased over time, while complications and length of stay decreased or remained unchanged. In this paper, we present findings from the qualitative evaluation of our ERAS implementation, an interview-based study with the programme’s champions. The study objective was to understand, from the champions’ perspectives and experiences, what influenced the success and sustainability of ERAS implementation in these centres. The Normalization Process Theory (NPT) [31] is used as an organizing framework to elucidate the individual and collective cognitive and social processes at work in ERAS programme implementation, with a view towards supporting sustainable change in surgical practice and patient care broadly (Table 2).

**Table 2:** Normalization Process Theory constructs and definitions

Construct	Definition
Coherence	The process and work of sense-making and understanding that individuals and organizations have to go through in order to promote or inhibit the routine embedding of a practice.
Cognitive participation	The process and work that individuals and organizations have to go through in order to enrol individuals to engage with the new practice.
Collective action	The work that individuals and organizations have to do to enact the new practice.
Reflexive monitoring	The work inherent in the informal and formal appraisal of a new practice once it is in use, in order to assess its advantages and disadvantages, and which develops users’ comprehension of the effects of a practice.

Reference [33]

## Methods

### Study design

A qualitative process evaluation was conducted from June to September 2014. The evaluation aimed to assess implementation quality and effectiveness for sustainable uptake of the ERAS programme among participating centres. Research ethics approval was obtained from each of the 15 participating sites. Consent was obtained from each participant.

#### Project leadership team

The project leadership team was comprised of a surgeon leader and principal investigator, two surgical lead champions, one lead champion in each of anaesthesia and nursing, and a project coordinator. Since 2006, these team members have advanced a quality initiative at the University of Toronto called Best Practice in General Surgery (BPIGS), of which the overall goal is to optimize care in general surgery. BPIGS itself is comprised of representative surgeons and anaesthetists from eight university-affiliated hospitals, including some of the individuals identified as champions for ERAS implementation. All members of BPIGS contributed to the development of the ERAS guideline (www.bpigs.ca). The project leadership team then designed and carried out the implementation by developing the protocol, presenting the guideline at participating sites, facilitating biweekly conference calls with nurses and monthly calls with surgeons and anaesthesiologists, moderating annual workshops where data and best practices were discussed, and acting as an all-purpose resource for participants.

### Settings

Fifteen hospitals in the province of Ontario participated. The sites were selected for inclusion by first, expressing interest to the funder to participate in the project and subsequently being approved by CAHO. Site characteristics are summarized in Table 3. The CEOs of each participating hospital formally signed onto the implementation project and in writing protected time for champion involvement. Sites varied in their prior experience with ERAS: eight sites had already begun ERAS programmes at the time of the project launch as a result of their BPIGS involvement. These sites began collecting data on ERAS patient outcomes between May and July 2013. The remaining sites had no prior ERAS experience; data collection in these sites began between July and November 2013. However, at some of these hospitals, ERAS-type care was already delivered though not in a bundle format and not identified as ERAS.

**Table 3:** Site characteristics for the study period May 2013–January 2015

Site	No. of enrolled patients	No. of participating surgeons
1	56	2
2	63	7
3	79	5
4	96	5
5	99	8
6	109	5
7	113	5
8	136	10
9	172	8
10	188	4
11	213	5
12	214	9
13	226	11
14	277	9
15	434	9

### Participants

For the evaluation, we aimed to recruit all participating hospital champions. This purposive sampling strategy targeted one nurse, one surgeon, and one anaesthesiologist from each site who together comprised the hospital’s local implementation team. Individuals became champions in their sites in different ways: the majority were asked (*n* = 19) or delegated (*n* = 17) to take the position, either because of prior interest, involvement with BPIGS, or fit with their clinical role; some champions volunteered (*n* = 8). Champions who were delegated the role reported being “volun-told” to take the position by a manager or senior hospital leader, in which case they did not feel they had the option to decline. This is differentiated from champions who were asked to consider the champion position and who felt they could refuse. The champion role description was to lead the implementation through stakeholder education and engagement; oversee local data collection, reporting, and auditing; and liaise with the project leadership committee. Champions were asked to attend two annual workshops and monthly (surgeon/anaesthesiologist) or bi-weekly (nurse/coordinator) teleconferences. For the evaluation, we also recruited study research coordinators as they worked closely with the champions and were considered part of the implementation team. In total, 58 participants were recruited for the evaluation (Table 4).

**Table 4:** Interview participants

Participant role	No. of interviews/eligible participants
Surgeon champions	15/15
Anaesthesiologist champions	14/15^a^
Nurse champions	15/15
Coordinators	14^b^/15^c^
Total	58/60

^a^One anaesthesia champion is on leave of absence

^b^At one site, both the former and current site coordinator were interviewed

^c^One coordinator was not available

### Data collection

Semi-structured interviews were conducted over the telephone or in person. All interviews were audio recorded and transcribed, lasting on average 33 min and ranging from 20 to 58 min. The interviews were conducted by a medical anthropologist with qualitative evaluation expertise (LGC) and an experienced nurse researcher (MM). LGC joined the leadership team to design and carry out the evaluation and was not well known to participants. MM was the nurse lead champion for the project and was known to many participants. To mitigate any potentially perceived biases or conflicts, MM did not interview any nurse champions for the evaluation. A common interview guide was used with open-ended questions exploring the local implementation processes and experiences. The interview guide was informed by the existing literature in guideline implementation, as well as researcher training and experience with qualitative interview design. Prior to use, the interview guide was reviewed and agreed upon by all authors whose combined expertise in guideline development and implementation in surgery contributed to its face validity. To ensure rigour in the data collection process, the interviewers met in person every 2 weeks throughout the data collection period to compare findings and modify the guide accordingly. During these meetings, the interviewers discussed the emerging findings from ongoing interviews and developed a common coding scheme using the constant comparison method [32]. The coding scheme was revisited, refined, and elaborated during each subsequent meeting. The interviewers’ respective experiences in implementation evaluation research and surgical nursing research contributed to the trustworthiness of the data collection process via investigator triangulation [33]. Each interviewer coded her own interview transcripts, though all interviews were jointly discussed. Discrepant opinions in the application of the codes were resolved by discussion until consensus was reached on code and category labels and content for all interviews. We used Nvivo10 for data management.

### Data analysis

An iterative inductive thematic analysis was initially conducted with data coded by both interviewers independently using an open coding process. Subsequent to the initial inductive data coding and categorizing, we turned to the NPT to provide an analytic framework through which to interpret and present our findings [32, 34, 35]. NPT offers a sociological framework that effectively accounts for the material, cognitive, and cultural components of implementation and sustainability at both individual and collective levels. NPT has been used as an explanatory model to examine and understand the enablers and barriers that emerge in complex implementation processes [34]. We selected the NPT because it is chiefly concerned with how new practices and processes are integrated, embedded, and sustained into routine practice. As discussion of these concepts emerged strongly in our data, the NPT provided an existing framework to organize and think through these findings.

Following our data-driven analysis, we used the well-established constructs within the NPT as sensitizing concepts to capture nuances in our interview data and organize them in a sensible manner. This was achieved by carefully reviewing the constructs against our existing data categories for alignment and fit. Given that the interview guide was not developed with the NPT constructs in mind, some components were not discussed by participants and were therefore not applicable in the final analysis presented here.

### Findings

Thematic findings are presented in accordance with the NPT. Additional supporting quotes illustrating participants’ experiences in ERAS implementation and alignment with NPT constructs are provided in Tables 5, 6, 7, and 8, respectively.

**Table 5:** Coherence—supporting quotes

Champion fit	Actually I was interested in the Fast Track before the others were interested. We started doing this at [hospital] with Surgeon X and Surgeon Y before everybody started doing it. I think we were probably doing it probably for about two or three years before everybody started. (Anaesthesiologist)
	Basically we started a similar programme a few years ago and that’s still in progress - Enhanced Recovery Colon Surgery. So it was called ERCS and we had a little bit of funding as well to start it in the hospital and there were a few uptake from other surgeons. It was difficult to convince them at that time. And so it became almost natural, when ERAS came in, they suggested that I take the lead on this one. (Surgeon)
Buy-in	The six surgeons who are seeing patients, they’re all engaged in this. I think one of the greatest things is that people know that ERAS is not a crazy thing, it’s structural. You can accommodate people. There are 10 interventions that you do, but if you cannot do ten, you can do only 5. But just do 5 and try to accommodate people and that way people feel happy with that. (Surgeon)
	Luckily it was easy to implement our part from an anesthesia perspective because the guidelines fell into what we do anyway and we do have the resources, the manpower, and the knowledge for the most part to implement these and the guidelines were no surprise to anyone. (Anaesthesiologist)
Resistance	I think that was one of the biggest challenges was feeding patients early because for so many nurses, they associated that with patients developing ileus. I think some of them still do. They feel that the patients who develop an ileus, it must be because we fed them early. (Nurse)
	There are a lot of people who are very critical or skeptical of the value of Gabapentin and are concerned that it makes the patient sleepier post-op. And you know, it’s kind of an issue of personal preference. I think the evidence for Lidocaine infusions is very strong and most people do as well but again, you just have to read the literature to know that. (Anaesthesiologist)
Team cohesion	Our surgeon champion’s great. I send him an email. He’s emailing me when he's on bloody holidays last week. I didn’t realize he was away. (Nurse)
	I think the interaction between the surgeon champion and nurse champion and myself have been very very good. I think the team has really helped, I think ERAS has really helped the teamwork. (Anaesthesiologist)
	The only challenge that I saw personally was trying to get our anesthesia champion interested enough to get his group onboard so that’s where the challenges were from my viewpoint. (Surgeon)

**Table 6:** Cognitive participation—supporting quotes

Community of practice	The network of all of the champions from the hospitals has been so instrumental in helping our hospital. The continued ongoing communication with the monthly phone calls for example. The website being fantastic, there’s just been so much support around this. (Nurse)
	The networking and the sharing of resources, I thought that was just absolutely fabulous. The people that were chosen from each hospital I think were really good champions. Very well-rounded, experienced, self-confident, not about, “look what I’ve done.” More like, “this is what we’ve done. Do you want it?”(Nurse)
Engagement strategies	Eventually I just met with several of the more resistant people to get a sense of what their concerns were and whatnot. So we’ve had meetings, we’ve had emails. A variety of different things to get people to buy into it essentially. It’s been a long process of that but eventually people have. (Surgeon)
	I go to most ORs and talk to the anesthesiologists to try and translate and clarify areas and make them understand what compliance with the program meant. (Anaesthesiologist)
Opportunities for co-creation	For me it was very important because it set the stage for partnership, so we had OT, PT, dietician involved. For each section, we had the ET nurses, we had frontline nurses, we also had the charge nurses. It was very very important because I wanted them to have the sense that they developed it. I’ve given you guidelines. How are we going to do it? It was very very important. Better buy-in. (Nurse)
	We got together and formed a work group of all the people who would be involved in implementing the various parts of the guideline. So this group was about 10 people, there was a dietitian, someone from physio, someone from OT, someone from nursing etc. Everyone who would have a stake in or in their workflow being changed and then over the next several months, we looked through the guideline and each piece worked on implementing their own, and me and the nurse person for that would essentially lead those meetings to try to get things implemented. (Surgeon)
Provide updates	I think it was about 3 months ago we got a report and I emailed the department the report and told them what our hospital number was. And then in the email I just mentioned some of the places we need to do a little bit better. (Anaesthesiologist)
	We did a follow-up series of lunch and learns as well as breakfasts where we actually presented the data to the different areas because it’s been about a year and a half or so that we’ve been involved in ERAS so we presented some of the data from the report that we got back. (Nurse)

**Table 7:** Collective action—supporting quotes

Chief support	I think you have to give some credit to the chief in Anesthesia. He accepted the ideas almost right away and [study PI] and I went to speak to the chief of all departments directly and he supported it. (Anaesthesiologist)
	It took a lot to get [name] onboard. He’s the Chief of Anesthesia. He kind of had the attitude of, which really pissed me off, sorry, “oh, we don’t need cheerleaders. We just need to do it. Like people will just adjust." Well, no, you need the education. People will have questions and answers and you know, giving opportunity to ask questions so they understand. I mean, I think that was a big big hurdle there. He was very difficult to get onboard. (Anaesthesiologist)
Systems integration	We’ve actually automated it so it appears on our OR schedule. Senior management asked our IT guys to associate ERAS procedures with the tag “ERAS.” So on our OR record, they show up as “ERAS rt hemicolectomy” as a flag to both surgical and anesthesia team that this patient is an ERAS patient. (Anaesthesiologist)
	The anesthesiologists aren’t unique to colorectal so it was a much bigger group of people to try and engage. You know, several months after we started, I'd be in the room with an anesthesiologist and they’d say, “so what’s this ERAS anyway?” So there was a piece of education there that was a bit harder to do. (Surgeon)

**Table 8:** Reflexive monitoring—supporting quotes

Use of data	Since we last got the ERAS report, we did a follow-up series of lunch and learns as well as breakfasts where we actually presented the data to the different areas, because it’s been about a year and a half that we’ve been involved in ERAS so we presented some of the data, as well as just thanked the staff for their contributions. (Nurse)
Need for audit and feedback	People need to see the impact of what they do. And that will be a challenge in that continuing to have information readily available to show the impact of what they’re doing and help them understand what they do makes a difference. I think that’s something that will make it sustainable. But that’s challenging because right now, we’re actively collecting data on these patients and that will eventually go away. (Surgeon)
Evidence of a culture change	I do think there’s been a culture shift. I think that’s something that really can take a long time. I don’t think we’re 100% there yet but I think we made some great strides in that way. Because that for me is one of the most important things is if you’re going to have sustainability you have to have people believe in the program, believe in the guidelines. (Nurse)
Normalization of ERAS	I want to actually get rid of the word ERAS completely just because I think it makes people think that there is something else other than enhancing someone’s recovery. There is nothing else. Every patient you’re trying to enhance their recovery. So there is no patient who shouldn’t be ERAS. (Surgeon)
	I think people don’t even think of it as a trial or a project anymore and I think the data’s already reflected that it’s been beneficial for our length of stay. So it’s not as though from our point of view, things are going to change. Everyone’s going to treat all colorectal surgery cases with an ERAS protocol at our hospital. I think people realize it’s the way it’s going to be. (Anaesthesiologist)

### Coherence: for whom and how does the ERAS programme make sense?

Many surgeon and anaesthesiologist champions were already aware of the ERAS principles at the time of implementation and had already adopted interventions in their practice. These participants viewed themselves to be the “logical” or “natural” person to take on the champion role. Nurse champions were mainly clinical nurse educators or coordinators with no prior knowledge of ERAS, except for one participant who had previously implemented a successful but short-lived ERAS nursing protocol. Despite the variability in their prior knowledge of what ERAS entailed, and how they came to be in the position, all but one champion described themselves as being the right fit for the role. They viewed it as a reasonable extension of their ongoing work to adopt best practices in surgical patient care. As described by one nurse champion,

In my Clinical Coordinator role, I was able to see patients through the whole surgical experience so it gave me some insights to the processes and experiences of patients. I already identified in my practice what can be done differently, what new, best practice is and can be implemented for these patients. And so that’s why this role came nicely integrated into the ERAS champion role. (Nurse)

Champions reported that some of their colleagues were easily accepting of the programme though they also met with individual-level resistance. Where buy-in was perceived to be easy, champions described the programme’s alignment with providers’ commitment to evidence-based practice, as well as an overall coherence with a department’s approach to guideline use. In departments where ERAS principles were not entirely new, acceptance of the programme was straightforward.

Everyone’s onboard. We were already pretty ahead of the curve in terms of postoperative pain management. We do have a lot of laparoscopic surgery so everyone did adopt the Lidocaine infusion. (Anaesthesiologist)

Champions attributed resistance to lack of agreement with the guideline pertaining to specific interventions that were a significant practice change. Among surgeons and nurses, resistance to the elimination of preoperative bowel preparation and early postoperative feeding were common which, as one participant stated “is probably going to require a retirement or two to change.” Among anaesthesiologists, resistance concerned the use of the analgesics Gabapentin and intravenous Lidocaine. A few senior providers were said to have “a firm belief that this is not going to work” and were therefore completely disengaged.

Champion teams described a collective sense of responsibility for the implementation. Although they worked independently to promote the programme within their disciplines, teams stayed connected to one another in order to problem-solve and address emerging barriers. Team cohesion was apparent in how champions offered one another support and expressed appreciation for one another’s efforts.

There were times where [anaesthesia champion] felt like she needed more support because I think there are more cultural issues in Anaesthesia where people don’t want to change. For a while, I think she felt like she was really on her own and so I would try to do things like ask someone in leadership the Department of Surgery to talk to somebody in leadership in Anaesthesia to be more supportive, to make her feel more supported. (Surgeon)

Fewer champions described poor team cohesion. Where they did, it occurred particularly in sites where the champion role was assigned, rather than being asked. This led to a sense of team fragmentation and champion turnover. In one such site, it was explained, “The nurse champion had always been told by their boss that this was essentially an add-on to what their full-time job was already.” Most champions regarded their own roles, and those of their co-champions, as invaluable, describing themselves as “the glue that holds the process and the team together” with a mandate to be “pushing and promoting” and “being a positive force within it”.

### Cognitive participation: by whom and how was a community of practice established?

A key component of the implementation was building a community of practice among the champions within their disciplines and across the participating centres. The leadership team facilitated this at a high level through organized opportunities for networking and sharing best practices. Daily and ongoing communication between the sites was enabled by a project listserv used mostly by the nurses and coordinators to share resources. Nurse champions in particular regarded the accessibility of the other ERAS centres, with varying degrees of experience, to enable their mutual success. Within the disciplines, these participants viewed others as their programme partners.

One thing unique to ERAS is you have your external partners. And we look forward to meeting as a group. And you know that there’s this community not just locally, but outside, who talks the same language, who shares the same challenges, and you can lean on them and seek their support. (Nurse)

All champions undertook concerted efforts to build relationships in their hospitals. This involved a tremendous amount of engagement work early in the implementation, as described by participants, to “build the capacity to really implement this programme and to sustain it.” A significant amount of time was therefore devoted to understanding current practices on the ground, raising awareness about the guideline, and reviewing the evidence with colleagues where necessary. All champions invested time in talking to people across their organizations, as one coordinator explained, “We had to do personal visits to pretty much every department.” Face-to-face meetings which facilitated relationship-building were described as especially time-consuming yet most crucial. This involved creating opportunities for colleagues to learn about and contribute to local implementation plans. Meetings were deliberately aimed at drawing other providers into the programme, accepting and integrating their ideas. Explicit strategies were used to avoid the sense of coercion or a top-down approach.

There was an educational component about why we do this, why are some of these components important that you may think are not important. But I also wanted to know quite frankly what they thought about it. I really wanted an open discussion where I was hoping that the nursing staff could feel that they could say no or add something in if they wanted so that right from the very beginning, they never felt like ERAS was coming upon them but they were creating ERAS. (Surgeon)

Subsequent to the initial launch, champions worked to build and sustain interest in the programme by formally and informally delivering updates to stakeholders on uptake and outcomes. Most teams did this using data reports provided by the leadership team, though some produced local reports on specific aspects of the programme targeting different groups. Where there was a large number of potential providers, such as in anaesthesia, champions made continuous efforts over several months to connect those who may have been missed in the early roll out. The engagement piece was therefore ongoing in order to capture every potential ERAS provider.

### Collective action: how was ERAS integrated into existing technological and social systems?

Champions were charged with building both the social and technological systems to optimize ERAS implementation. Many sought support from their respective departmental leaders for establishing confidence in the interventions. Several champions reported the influential role of departmental chiefs, particularly in anaesthesia; lack of division head support was felt to impede uptake.

Some of the things that Anaesthesia was asked to adopt were much more out of their day to day routine and their comfort level than what we were asking surgeons to do. Our Chief of Anaesthesia had to step in a little bit and advocate for the ERAS programme so that things have eventually straightened out. (Surgeon)

More and more people are using Lidocaine infusions but not everybody agrees with them, most notably the Chief of our department. And I felt that in some ways I haven’t had the support that I had hoped I would get for implementation of this guideline. (Anaesthesia)

Electronic systems integration was a key undertaking with which many champions struggled. Participants described such processes as the institutional approval of new order sets as moving at a “glacial” pace. Yet most champions reported eventual success integrating ERAS into computer order entry systems such that patient identification and ERAS preoperative orders were automated. Operationalizing this successfully was attributed to finding places within existing systems where ERAS would both be seen and be seamlessly integrated.

I had the computer system add a flagging so that we could identify the patients correctly starting from the surgeon’s office and I think that was key, in combination with the stickers, having the patient be flagged as ERAS from when they came to the hospital all the way through. (Coordinator)

Complete integration with staff scheduling proved to be challenging in anaesthesia where potential provider numbers ranged from 20 to over 100. Anaesthesiologists may therefore hear about ERAS, but may not see an ERAS patient for months at a time. Anaesthesia champions tried some restructuring strategies to overcome this barrier, identifying a small group of providers who would do ERAS cases. Those who tried this found it not to be sustainable, as one champion explained, “because lot of anesthesiologists had complained about having a special group of ERAS anesthesiologists.” Uptake in anaesthesia was perceived to be slower as a result.

### Reflexive monitoring: how do champions assess the implementation effectiveness?

Participants considered the availability and use of data to be a main driver of effective implementation as it allowed everyone to see concrete results of their efforts and allowed comparison to other centres. In one site, a nurse champion described an ERAS display board with “the entire data sheet related to us with a circle around our data.” A majority of champions found that sharing the data reports helped overcome skepticism and resistance.

The most valuable influence on my division was probably the first time we went over the report. And I was able to say ‘Look at what’s happening with our patients. Look at how we are comparing to other institutions. Look at what we’re doing’. That had a very significant impact and I even had some surgeons who were actually quite obstructive in this whole process ask me for a copy of the report and they’re engaged now. (Surgeon)

Participants expressed concern that without the audit and feedback, which was only supported financially until the end of the funded project, sustainability was threatened. Though hospitals had incorporated the ERAS programme into their electronic systems, many believed the clinical indicators were needed to keep momentum.

I think if the data collection stops, I'm afraid that it won’t be sustainable. I think part of what’s keeping it going is the notion that somebody’s watching and the numbers are being gathered. (Anaesthesiologist)

Champions also used more informal, ad hoc means to continuously review uptake and reinforce the programme. They became known locally for their champion roles and were able to periodically address gaps in practice with individual providers, as described by one surgeon: “I get usually negative feedback from the nurse unit leaders if someone is not getting the right diet. So I’ve become sort of the ERAS police at this hospital for better or for worse.”

Many participants described a sense of satisfaction with the degree of buy-in they had achieved, and the extent to which they believed ERAS was embedding. This was described as evidence of a slow but steady culture change; one that was spreading to other specialties in their centres.

I can’t see how it hasn’t shifted culture and people are still looking to shift more. I think there has been a tremendous change. And it’s moved into other specialties and those other specialties are starting to wake up. (Anaesthesiologist)

As part of the culture change, participants felt that ERAS would be reconfigured locally as the standard of care and that people would stop using the ERAS label. As one participant stated, “You don’t hear the word ERAS a lot anymore, but it’s natural because it does become the standard of care, then there’s nothing to talk about.” This normalization of ERAS was believed necessary among healthcare teams and hospital administration for sustainable practice change. In this regard, all participating organizations were described by participants as “supportive”, though no champion was granted any protected time or compensation for their role, as was written in the contracts between the hospitals and funder. A number of participants therefore worried that their organizations’ perception of ERAS as a one-time initiative would undermine its sustainability.

I think the culture change will be sustained. I think the patients will still get the ERAS-type clinical service but obviously I don’t think our hospital will fund a nurse to run ERAS. I don’t see that coming, not in a million years. (Anaesthesiologist)

## Discussion

Through qualitative interviews with project champions, we sought to gain insight to the uptake and sustainability of ERAS implementation for elective colorectal surgery. Using the NPT, we have identified a number of implementation enablers: the belief of project champions in the value of the programme, the fit and cohesion of champions and champion teams, a bottom-up approach to stakeholder engagement targeting relationship building, receptivity and support of division leaders, and the normalization of ERAS as everyday practice. Technical enablers included effective integration with existing clinical systems and using performance outcomes to report to hospital stakeholders. The main barriers reported by champions in this study were provider resistance to practice change, poor administrative support, and large numbers of anaesthesia providers at some sites. There was an overall optimism that ERAS implementation would be sustained, accompanied by concern about long-term organizational support.

The NPT framework has been useful for pinpointing the influential cognitive and social processes that enabled ERAS implementation. First, we found that the dedication with which champions in this project either accepted or assumed their roles was remarkable. Though more than a third had been delegated the position, all but one believed themselves to be well-equipped and appropriately selected to fulfil its responsibilities. This one individual, who was is in a manager role, felt too removed from direct care to advance the initiative as well as too short of time to commit to the project. While the literature on the most effective champion selection method is inconclusive [36–38], we have found that the degree of individual coherence is a very strong driver. In this study, the champion role was successfully cultivated among delegated individuals with certain characteristics and under certain conditions: if there was a real belief in the value of the programme, if the individual had the awareness and ability to build the necessary interprofessional relationships, and if s/he could effectively communicate and negotiate with colleagues. Given that no formal training was provided, coherence with champions’ existing beliefs and abilities was essential. In sites where initial champion turnover was experienced, this may be explained by a lack of coherence with individual beliefs and skills. In addition to individual coherence, implementation team cohesion and department-level support were found also to be strong contributors to success. As a result of these supports, champions were able to effectively adapt the protocol to their local culture and systems. Each implementation team was encouraged to figure out how to best make ERAS “fit” their organization. The implementation programme was not entirely prescriptive to that end and there was, consequently, variability in how the programme was ultimately operationalized within the centres’ systems. Our findings suggest however that, on the ground, champions’ beliefs in the importance of the programme and their ability to adapt the programme to suit the variable local contexts enabled their success. These findings are aligned with those of other multisited implementation studies in which similar champion characteristics and contexts are found to be necessary mechanisms for driving local practice change [39, 40].

Second, the development of a community of practice was an explicit part of the knowledge translation strategy through which champions were guided and supported by the project leadership team. As the initiative’s “vertical core” [41], the leadership team established sufficient trust with champions, empowering them to build networks within the hospitals using whatever means they deemed suitable and developing whatever tools necessary. The external and internal partnership building were key and also strategic, so as not to impose ERAS but to co-create it from the ground up. This relational work, as framed in the NPT, is deceptively complex as it involves convincing others that this is a legitimate improvement programme worth participating in without devaluing their current practice and beliefs [40]. The interprofessional and interdepartmental relationships the champion teams established appeared to lay an important foundation for accepting changes and the data reports as meaningful and embedding ERAS into everyday practice.

Finally, our findings revealed that successful ERAS implementation requires a movement over time from very high to very low visibility within the consciousness of an organization, without completely disappearing. ERAS champions had to initially make ERAS highly visible to all relevant stakeholders by talking extensively about the project, flagging patients, labelling orders, and visually integrating ERAS into existing technological systems. Data reporting and display boards provided additional visualization which champions found effective for buy-in and reinforcement. This operational work constituted the architecture of the programme on the ground which required significant time and resource investment. It is likely to contribute to the long-term sustainability of the programme. Over time, however, champions described the need for ERAS as a special programme to essentially disappear if it was to become normalized practice. To this end, the programme must work against it own “projectness” [40], that is, its status as a project with funded, time-limited resources. Champions’ concerns about the current and future support of ERAS at the organizational level represented this tension between ERAS visibility and invisibility which is at once essential and threatening to its sustainability. The continuity of the audit and feedback process was believed to potentially mitigate the threat of total invisibility, though most champions worried that organizational resources would not be provided long term; this concern was reinforced by the absence of champion compensation or protected time from the organization. Many champions therefore remained optimistic that the programme would be sustained through their own and others’ interests, while also expressing concern about the loss of the audit system which could provide just the right amount of organizational visibility for the future.

This study has demonstrated that the NPT can effectively be used a thinking tool, to organize and interpret the meaningfulness of the many interlinked and complex processes that are involved in large-scale healthcare implementation. Our study also has limitations. While qualitative interviews are useful for understanding participant experiences, real-time ethnographic fieldwork in the hospital settings would provide richer insights to site-specific contextual factors that may have influenced implementation. This type of real-time evaluation however is challenging [41]. In addition, findings of this study may not be generalizable to non-academic or community settings implementing ERAS. Finally, only with longitudinal data collected post-implementation will we know the extent to which ERAS is actually sustained in participating centres, and the potential impact of champion attrition or turnover on long-term sustainability of the ERAS initiative.

## Conclusions

Successful ERAS implementation is achieved by a complex series of cognitive and social processes which previously have not been well described. The NPT offers a framework for identifying specific processes that enable or impede implementation, as well as areas for to target for future implementation efforts. This analysis has demonstrated the importance of champion coherence, external and internal relationship building, and the strategic management of a project’s status as a “project”, as important to the uptake and normalization of ERAS. Long-term sustainability of ERAS under these conditions is an area for further research.

## Abbreviations

ERAS: enhanced recovery after surgery
NPT: Normalization Process Theory
